# A modular class-aware workflow for small RNA sequencing analysis using mouse sperm as a case study

**DOI:** 10.1080/17501911.2026.2725425

**Published:** 2026-09-06

**Authors:** Da Lu, Huan Liao, Tishtar Daruwalla, Anthony J. Hannan

**Affiliations:** a Florey Institute of Neuroscience and Mental Health, Parkville, VIC, Australia; b Florey Department of Neuroscience and Mental Health, University of Melbourne, Parkville, VIC, Australia; c Department of Anatomy and Physiology, University of Melbourne, Parkville, VIC, Australia

**Keywords:** Small non-coding RNA analysis, miRNAs, piRNA clustering, tsRNAs, rsRNAs

## Abstract

**Background:**

Small RNA sequencing analysis is challenging because RNA classes differ in biogenesis, sequence redundancy, genomic organization, and annotation reliability. Integrated workflows accommodating these constraints remain limited, particularly for fragment-level and cluster-level analysis.

**Methods:**

We present a reproducible, containerized, class-aware workflow for small RNA sequencing analysis, using mouse sperm as a case study. The workflow combines standardized preprocessing with complementary annotation and quantification strategies for microRNAs (miRNAs), transfer RNA-derived small RNAs (tsRNAs), ribosomal RNA-derived small RNAs (rsRNAs), and PIWI-interacting RNA (piRNA)-enriched genomic clusters. Using sperm small RNA data from offspring of lipopolysaccharide (LPS)-exposed male mice, we compared integrated-reference mapping, multi-class annotation, fragment-level tsRNA profiling, and genome-based piRNA cluster analysis, with custom modules for locus-aware harmonization and condition-specific cluster analysis.

**Results:**

Integrated-reference mapping aligned 88.17% of reads and retained 690 features after filtering. It identified 11 differentially expressed miRNAs between LPS and controls, while other classes showed limited signal. Fragment-level profiling improved tsRNA resolution. piRNA cluster analysis identified 958 control and 940 LPS clusters, with 18 control-specific and no LPS-specific clusters.

**Conclusion:**

This workflow supports transparent, reproducible, class-aware interpretation of small RNA sequencing data while emphasizing cautious interpretation of piRNA-enriched signals from total small RNA sequencing.

## PLAIN LANGUAGE SUMMARY

Small RNA molecules are found in many tissues and biological samples. In sperm, these molecules may carry information about a father’s health or environmental exposures, but they are difficult to study because each RNA type has different features. We developed a workflow that uses a suitable analysis method for each RNA type. We tested the workflow using sperm from adult male mice whose fathers had received a bacterial substance that activates the immune system. The workflow detected changes in several types of small RNA and showed that some require more specialized analysis than others. For one RNA type, called PIWI-interacting RNA (piRNA), the workflow examined groups of signals arising from the same regions of DNA. These were treated as candidate piRNA-related signals for comparison between groups. The workflow provides a consistent approach for analyzing small RNA sequencing data and is designed to be adaptable to other biological samples and research settings.

## 1. Introduction

Small non-coding RNAs (sncRNAs) constitute a diverse class of regulatory molecules that modulate gene expression at the post-transcriptional level and contribute to epigenetic regulation across many biological processes [1]. Among sncRNAs, microRNAs (miRNAs) are ~19–24 nucleotide RNAs that guide Argonaute proteins to complementary target transcripts, resulting in mRNA degradation or translational repression [2,3]. PIWI-interacting RNAs (piRNAs) are longer small RNAs, typically 21 to 33 nucleotides in length, that are associated with PIWI proteins to suppress transposable elements and preserve genome integrity [4,5]. More recently, tRNA-derived small RNAs (tsRNAs) have emerged as an additional regulatory class generated by specific cleavage of precursor or mature transfer RNAs [6–8]. These fragments span a broad size range, from approximately 18 to 40 nucleotides, and include 5 prime, 3 prime, internal fragments, and longer tRNA halves [6–8]. Importantly, tRNA-derived fragments are heterogeneous regulatory molecules rather than interchangeable products of their parental tRNAs, as their cleavage positions, abundance, and biological activities vary substantially across cellular and physiological contexts [9]. This heterogeneity highlights the importance of retaining fragment-level information rather than relying solely on parental tRNA identity.

Beyond these major classes, ribosomal RNA-derived small RNAs (rsRNAs) and Y RNA-derived small RNAs (YsRNAs) are increasingly recognized as regulated components of small RNA populations [10–12]. Although many YsRNAs and rsRNAs arise from RNA processing or degradation, their recurrent size distributions and condition-associated abundance patterns have motivated investigation into potential regulatory roles [10–12]. In the male germline, the sncRNA landscape is extensively remodeled during spermatogenesis and epididymal maturation, with tRNA- and rRNA-derived fragments becoming prominent components of mature sperm [13]. Within this dynamic landscape, sperm tsRNAs have been reported to respond to environmental and dietary exposures and have been implicated in male fertility, embryo quality, early development, and paternal epigenetic inheritance [14].

Consistent with these observations, accumulating evidence indicates that exposure-associated alterations in sperm small RNAs can influence post-fertilization embryonic gene expression and early developmental programs [15–18]. Such changes may contribute to intergenerational epigenetic inheritance, defined here as the transmission of environmentally induced phenotypic effects to offspring through germline epigenetic information without alterations to the underlying DNA sequence. Studies across paternal environmental and physiological challenge models have further associated small RNA alterations with metabolic, immune, and behavioral outcomes in offspring [19–21]. Together, these findings underscore the need for analytical frameworks that can accurately and reproducibly profile multiple sncRNA classes while accounting for their distinct biogenesis, sequence characteristics, genomic organization, and levels of annotation.

High-throughput small RNA sequencing enables unbiased profiling of heterogeneous sncRNA populations, yet downstream analysis remains challenging due to the distinct biogenesis pathways, sequence properties, and genomic organization of each RNA class. miRNAs and rsRNAs are typically quantified using transcript-based annotations, whereas tsRNAs require specialized handling to address extensive tRNA redundancy and post-transcriptional modifications. In contrast, piRNA-producing loci are commonly organized as genomic clusters rather than conventional transcript units [22]. Therefore, cluster-level analysis of piRNAs based on genomic coordinates should be interpreted as operational proxies that require cautious reporting and interpretation.

In practice, comprehensive small RNA-seq analysis often requires combining multiple tools and reference databases. This can improve coverage across RNA classes but also introduces ambiguity when reads map to multiple small RNA categories, when different databases provide inconsistent annotations, or when class-specific biological properties are not considered during quantification. To avoid these challenges, many studies focus on a single RNA class or perform manual, class-specific workflows, which limit reproducibility and integrated interpretation. Although several established tools exist for individual sncRNA classes, few workflows provide coordinated, class-aware analysis across miRNAs, rsRNAs, tsRNAs, and piRNAs while respecting their distinct analytical constraints, particularly for piRNA cluster-level interpretation and tsRNA fragment resolution.

Here, we present a modular and containerized class-aware workflow for small RNA-seq analysis, designed to support reproducible profiling of miRNAs, tsRNAs, rsRNAs, and piRNA-enriched genomic clusters. Rather than proposing a new small RNA discovery algorithm, this framework integrates established tools with standardized processing, class-specific quantification strategies, and explicit reporting checkpoints, while introducing custom downstream modules for locus-aware piRNA cluster harmonization, cluster-associated read quantification, and condition-specific non-overlapping cluster interpretation. Using mouse sperm small RNA-seq data from a paternal immune activation model as a case study, we compare complementary analytical configurations and illustrate how class-aware analysis can improve transparency, reproducibility, and biological interpretation. Although demonstrated using sperm, the modular workflow can be adapted to small RNA-seq datasets from other tissues and biological contexts using appropriate reference annotations and class-specific configurations.

## 2. Materials and methods

### 2.1. Pipeline overview

An integrated and modular analysis pipeline was developed to support comprehensive small RNA sequencing analysis with explicit class-aware quantification and transparent downstream integration. The computational structure is not restricted to sperm and can be configured for other tissues by substituting appropriate organism-, tissue-, and RNA-class-specific references. The pipeline accommodates the distinct biogenesis mechanisms and regulatory architectures of major small RNA classes while maintaining standardized preprocessing and reproducibility across datasets. Raw small RNA sequencing reads undergo uniform quality control, adapter trimming, and length filtering to remove technical artifacts and degraded RNA fragments while retaining biologically relevant regulatory small RNAs. Depending on the analytical objective, the workflow uses complementary but statistically independent branches. The integrated-reference branch provides the primary feature-level analysis for miRNAs and rsRNAs, tDRmapper provides fragment-level tsRNA analysis, and proTRAC provides genomic piRNA-cluster analysis. For these class-specific analyses, tDRmapper and proTRAC apply read-length and feature-definition criteria before quantification to restrict downstream testing to defined tRNA-derived fragments and candidate piRNA-enriched clusters, respectively. Count matrices generated by these branches are analyzed independently and are not pooled or summed in a common differential-expression model.

Downstream analysis are performed in a class-aware manner, enabling appropriate statistical modeling and interpretation tailored to each RNA type. For piRNAs, *de novo* genomic clustering and cluster-level quantification are emphasized over individual-level analysis. The pipeline further supports optional downstream integration with phenotypic data through statistical and machine learning frameworks. This design provides a flexible yet standardized end-to-end workflow for small RNA sequencing analysis, enabling reproducible quantification, transparent exploratory prioritization, and extensibility to diverse experimental contexts.

### 2.2. Sample data preparation

Small RNA sequencing datasets were generated from sperm isolated from the distal cauda epididymides of male C57BL/6J mice. The paternal immune activation paradigm, mating design, and sperm isolation procedures were conducted as previously described [19]. Briefly, eight-week-old males received a single intraperitoneal injection of lipopolysaccharide (LPS; 5 mg/kg; Salmonella typhosa, Sigma-Aldrich, Cat. #L6386-100MG), with saline-treated males serving as controls. Sperm used for small RNA sequencing were collected from adult male offspring at 8 weeks of age to assess germline small RNA profiles in the subsequent generation independent of direct inflammatory exposure. For each treatment group, five biological replicates were prepared, each comprising pooled sperm from three randomly selected males to increase RNA yield. Total RNA was extracted using the QIAGEN miRNeasy Mini Kit (Cat. #217004) according to the manufacturer’s instructions, followed by DNase treatment using the QIAGEN RNase-Free DNase Set (Cat. #79254). RNA concentration was measured using the Qubit RNA Broad Range Assay Kit (Cat. #Q10210) on a Qubit 4 Fluorometer. RNA integrity and potential somatic cell contamination were assessed using the Agilent 4200 TapeStation System with RNA ScreenTape reagents (Cat. #5067–5576 to #5067–5578). Small RNA libraries were prepared using the NEXTflex Small RNA v4 library preparation kit with bead-based size selection and sequenced at the Australian Genome Research Facility on an Illumina platform using a 300-cycle workflow. This conventional ligation-based preparation did not include enzymatic end-repair or demethylation pretreatment. Consequently, the resulting libraries may underrepresent small RNAs bearing termini that are incompatible with adapter ligation or internal modifications that interfere with reverse transcription. Sequencing yielded an average of 76,188,683 raw reads per biological replicate, with 48,395,643 reads retained following adaptor removal. Adapter trimming was performed using Trim Galore! (v0.6.7) [23], read quality was evaluated with FastQC [24], and summary metrics were aggregated using MultiQC [25]. Quality filtering was performed to ensure that bases across all samples exceeded a Phred quality score of 20.

### 2.3. External validation dataset

To benchmark the workflow against an independent published dataset, sperm small RNA-seq FASTQ files from Kleeman et al. [26] (NCBI SRA BioProject PRJNA1335862) were reanalyzed using the current pipeline. The dataset comprised four SARS-CoV-2 and five mock-control biological replicates, each generated from sperm pooled from three mice. Although these data had previously been analyzed using an earlier workflow implementation, the present validation applied the updated integrated-reference, SPORTS1.1, tDRmapper, and proTRAC modules using the filtering and differential-expression criteria described above. piRNA clusters were additionally compared between groups by genomic overlap to identify group-specific loci. The results were included in **Supplementary Figures S1–S2**.

### 2.4. Integrated reference-based small RNA profiling

**Figure 1.**
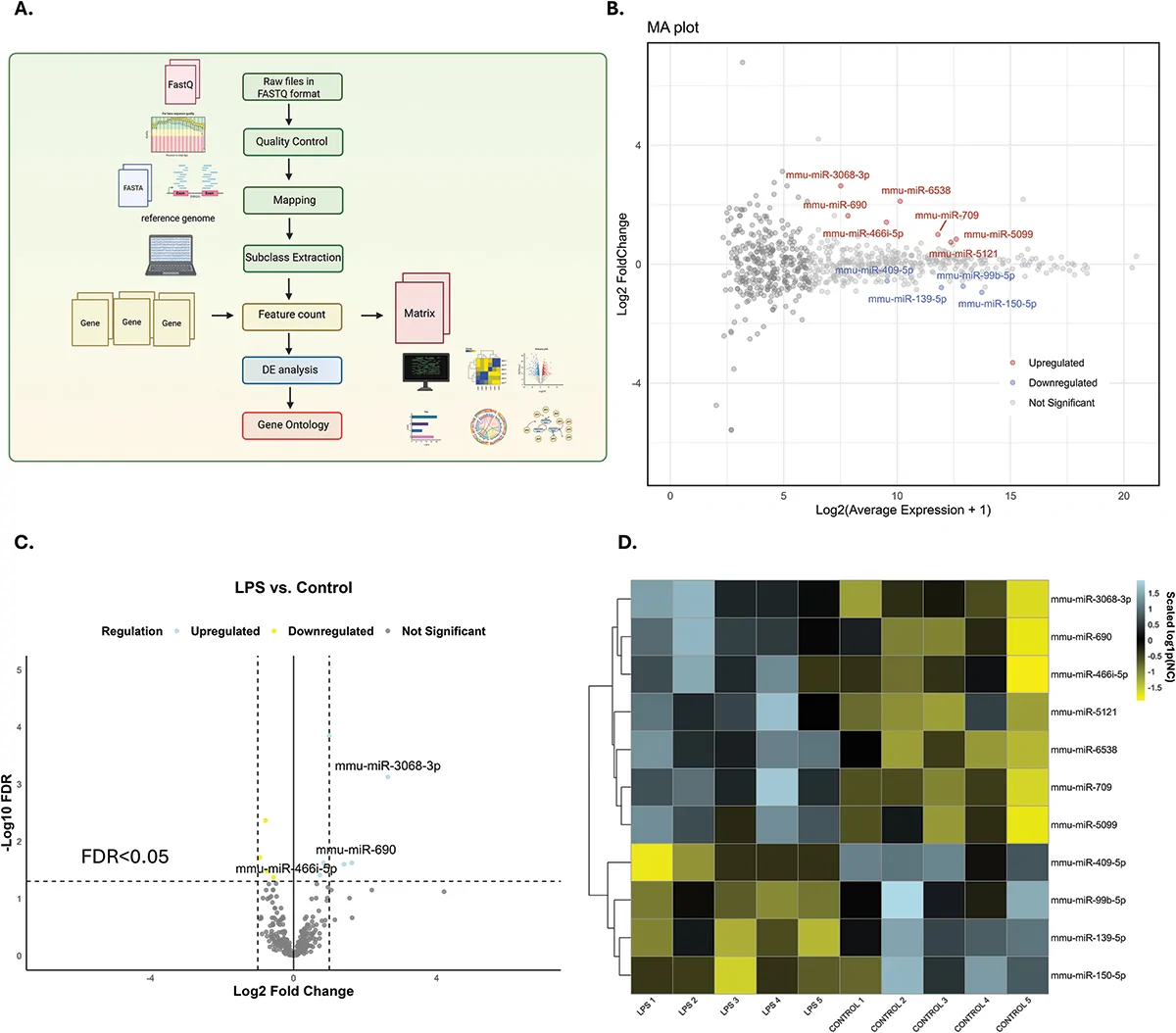
Multi-class small RNA analysis with an integrated reference framework. (A) Schematic workflow diagram outlining the analysis pipeline, including major processing modules and data flow from raw sequencing input to downstream differential expression analysis. (B) MA plot showing differential expression of small RNAs in sperm from adult male offspring of LPS-treated fathers versus saline controls. Points represent individual small RNAs plotted by log_2_ fold change against mean normalized expression, with significant features highlighted (FDR < 0.05). (C) Volcano plot depicting all detected small RNAs in sperm from adult male offspring of LPS-treated fathers versus saline controls. log_2_ fold change is shown on the x-axis and −log_10_ false discovery rate (FDR) on the y-axis. The horizontal dashed line denotes an FDR threshold of 0.05. Small RNAs exceeding the significance threshold (FDR < 0.05) are highlighted in yellow (downregulated) or light blue (upregulated). (D) Heatmap showing expression levels of differentially expressed small RNAs in sperm from adult male offspring of LPS-treated fathers versus saline controls. Values represent scaled log1p-transformed normalized counts across five biological replicates per group. The color scale indicates relative expression levels. Panel A was created in BioRender. Lu, D. (2026) https://BioRender.com/y9j4d6q.

All small RNA sequencing libraries underwent quality-controlled preprocessing prior to alignment and quantification. Cleaned reads were aligned using the Subjunc aligner from the Rsubread package (v2.12.3) [27] against an integrated reference index comprising mouse miRNA sequences from miRBase (v22.1; https://mirbase.org/ftp.shtml), transfer RNA sequences from GtRNAdb release 20 (https://gtrnadb.ucsc.edu/genomes/eukaryota/Mmusc10/Mmusc10-seq.html), and ribosomal RNA sequences including 5S, 16S, 18S, and 28S derived from RNAcentral (v24; https://rnacentral.org/), SILVA (v138.2; https://www.arb-silva.de/), and GENCODE GRCm39 (https://www.gencodegenes.org/mouse/) (Figure 1(A)). On average, 88.17% of reads per sample were successfully aligned to the composite index (weighted average mapping rate = 88.29%). Feature-level read counts were summarized using Rsubread’s featureCounts function, and RNA subtype annotation was assigned based on miRBase for microRNAs, GtRNAdb for transfer RNAs, piRNAdb v1.7.6 (https://www.pirnadb.org/) and piRBase (https://www.pirbase.org/) for PIWI-interacting RNAs, and RNAcentral, SILVA, and GENCODE for ribosomal RNAs. Differential expression analysis was conducted using DESeq2 (v1.40.2) [28], retaining features with a minimum count of 10 in at least three samples, resulting in 690 retained features from an initial set of 1978. Raw count data were processed using the internal size-factor normalization procedures implemented in DESeq2, and statistical significance was assessed using a false discovery rate threshold of 5%. Differential expression results were visualized using MA plots, volcano plots, and heatmaps generated with ggplot2 (v3.4.4) [29] and pheatmap (v1.0.12) [30]. For heatmap visualization, DESeq2-normalized counts were log1p-transformed and then row-scaled within each small RNA feature so that the heatmap displays relative, feature-wise scaled expression rather than absolute counts. The integrated reference-based framework was used for broad feature-level profiling of annotated small RNA classes, but it was not considered suitable for robust differential analysis of piRNAs or tsRNAs. piRNAs primarily function as genomic clusters instead of independent transcript units, rendering feature-level quantification of individual annotated piRNAs biologically and statistically inappropriate [22]. Similarly, tsRNAs originate from highly redundant tRNA loci with shared sequence motifs and multiple overlapping fragment types, leading to ambiguous read assignment and compositional confounding when analyzed alongside other small RNA classes within a unified reference model [31]. For this reason, downstream differential analysis of piRNAs and tsRNAs was performed using dedicated class-specific workflows rather than the integrated reference-based feature table.

### 2.5. Class-specific small RNA profiling using SPORTS

**Figure 2.**
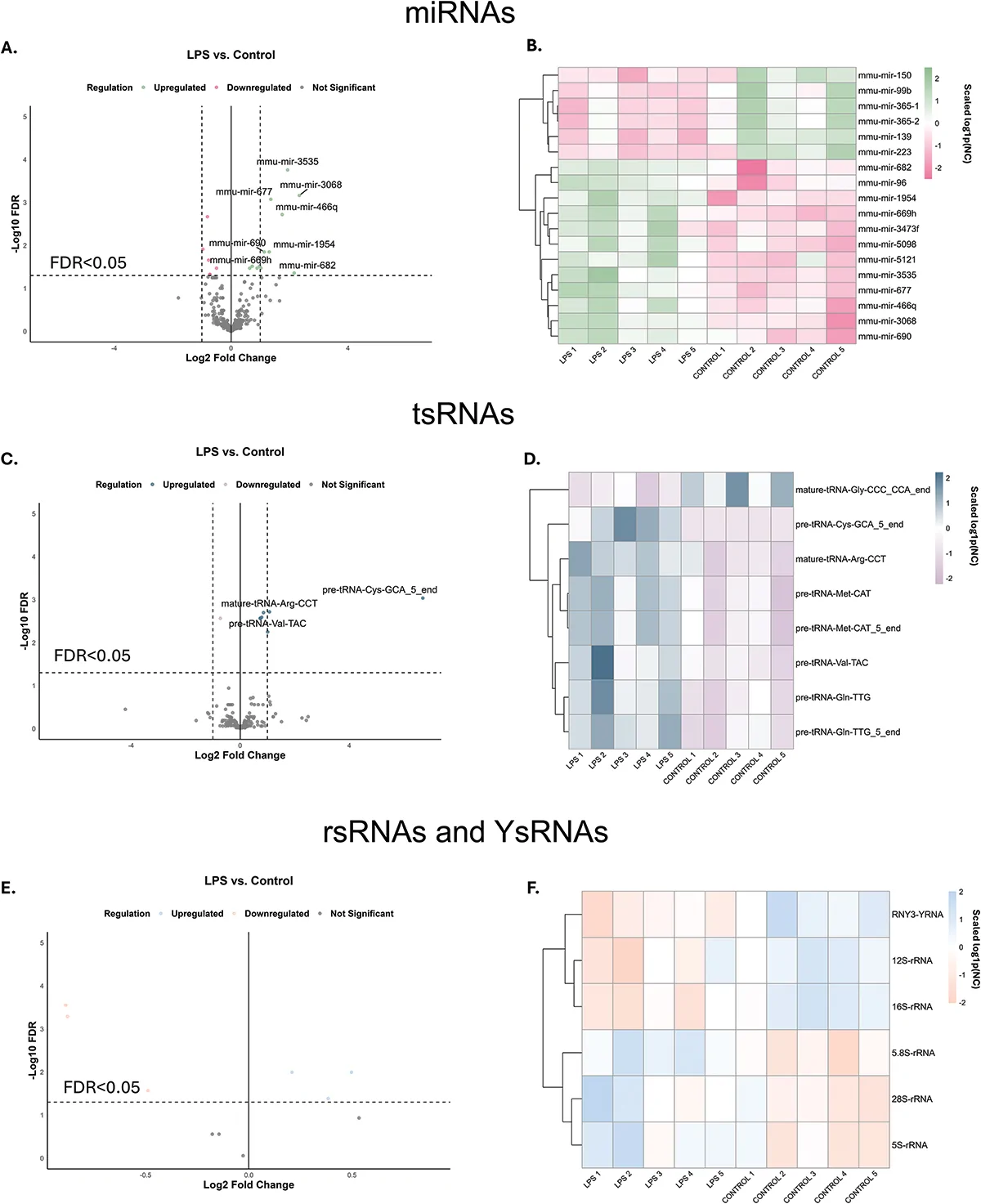
Class-specific small RNA analysis using SPORTS. (A) Volcano plot depicting all detected miRNAs in sperm from adult male offspring of LPS-treated fathers versus saline controls. Log_2_ fold change is shown on the x-axis and −log_10_ false discovery rate (FDR) on the y-axis. The horizontal dashed line denotes an FDR threshold of 0.05. Small RNAs exceeding the significance threshold (FDR < 0.05) are highlighted in pink (downregulated) or light green (upregulated). (B) Heatmap showing expression levels of differentially expressed miRNAs in sperm from adult male offspring of LPS-treated fathers versus saline controls. Values represent scaled log1p-transformed normalized counts across five biological replicates per group. The color scale indicates relative expression levels. (C) Volcano plot depicting all detected tsRNAs in sperm from adult male offspring of LPS-treated fathers versus saline controls. Log_2_ fold change is shown on the x-axis and −log_10_ false discovery rate (FDR) on the y-axis. The horizontal dashed line denotes an FDR threshold of 0.05. Small RNAs exceeding the significance threshold (FDR < 0.05) are highlighted in purple (downregulated) or dark green (upregulated). (D) Heatmap showing expression levels of differentially expressed tsRNAs in sperm from adult male offspring of LPS-treated fathers versus saline controls. Values represent scaled log1p-transformed normalized counts across five biological replicates per group. The color scale indicates relative expression levels. (E) Volcano plot depicting all detected rsRNAs and YsRNAs in sperm from adult male offspring of LPS-treated fathers versus saline controls. Log_2_ fold change is shown on the x-axis and −log_10_ false discovery rate (FDR) on the y-axis. The horizontal dashed line denotes an FDR threshold of 0.05. Small RNAs exceeding the significance threshold (FDR < 0.05) are highlighted in orange (downregulated) or sky blue (upregulated). (F) Heatmap showing expression levels of differentially expressed rsRNAs and YsRNAs in sperm from adult male offspring of LPS-treated fathers versus saline controls. Values represent scaled log1p-transformed normalized counts across five biological replicates per group. The color scale indicates relative expression levels.

SPORTS1.1 (Small RNA-seq Portal for Annotation and Expression Profiling) was used as an alternative multi-class annotation and compositional framework for cross-class comparison rather than as the primary inferential analysis [32,33]. For Mus musculus samples, reference databases included genome assembly mm10 (UCSC), mature miRNA sequences from miRBase release 21, a combined mouse rRNA database (18S, 28S, 5.8S, 5S, 45S), mature tRNA sequences with post-transcriptional CCA additions from GtRNAdb (mm10), piRNA sequences from piRBase, additional non-coding RNAs from Ensembl release 89 (GRCm38), and small RNA families from Rfam version 12.3. All references were indexed using Bowtie version 1.3.1 [34]. Following adapter trimming and quality control, cleaned reads (15-45 nt) were aligned with a maximum of one mismatch to ensure annotation specificity [32]. Class-specific read counts were extracted from SPORTS summary outputs to construct independent count matrices for miRNAs, tsRNAs, and rsRNAs/YsRNAs, excluding subtotal entries to prevent redundant counting. Matrices were analyzed separately using DESeq2 with consistent low-count filtering (minimum 10 reads in at least three samples) and FDR control (Benjamini-Hochberg adjusted *p* < 0.05). Class-specific differential-expression results were visualized using volcano plots and heatmaps in Figure 2. Its assignments are reference-order dependent and mainly provide broad parental or reference-level annotations for tsRNAs, rsRNAs, YsRNAs, and piRNA-like reads rather than the fragment-level or genomic cluster-level resolution required for the primary analyses.

Relative sncRNA class composition was additionally assessed using the per-sequence SPORTS annotation output (**Supplementary Figure S3**). For each sample, collapsed sequences were assigned to one non-overlapping class: miRNA, tRNA-derived small RNA, rRNA-derived small RNA, YRNA-derived small RNA, piRNA-like, or other/ambiguous. Reads were summed for each class and divided by the total clean SPORTS output reads for that sample to calculate the relative contribution of each major sncRNA class. Group-level differences in overall class composition were assessed using an exact permutation test based on a pseudo-F statistic applied to centered log-ratio-transformed class proportions.

FUSION is a family-level integration approach designed to improve the robustness of small RNA analysis in low-replicate datasets [35]. The processed class-specific read tables from both the LPS experiment and the published paternal SARS-CoV-2 validation dataset were therefore additionally evaluated with FUSION using the same sample groupings as the SPORTS analyses. FUSION integrates small non-coding RNA signals at the parental RNA family level, generating family-level summaries for miRNAs, tsRNAs, rsRNAs, YsRNAs, and other small non-coding RNAs. In the LPS dataset, FUSION_ms returned as significant 168 miRNA, 22 tsRNA, 7 rsRNA, 2 YsRNA, and 3 other small non-coding RNA features defined at the parental-RNA level. In the published paternal SARS-CoV-2 dataset, FUSION_ms returned as significant 115 miRNA, 17 tsRNA, 7 rsRNA, 1 YsRNA, and 3 other small non-coding RNA features defined at the parental-RNA level. However, FUSION was not used to replace the primary DESeq2-based analyses because it changes the statistical unit being tested. The primary models evaluated predefined feature-level, fragment-level, or locus-level count tables, whereas FUSION integrates unique sncRNA species according to their parental RNA annotations to test family-level differential abundance. Replacing the primary models with FUSION would therefore shift the interpretation from feature-, fragment-, or locus-level differential expression to parental RNA-family-level differential abundance. Accordingly, these outputs were treated as a complementary sensitivity analysis and were not combined with the primary integrated-reference, tDRmapper, or proTRAC differential-expression models because they represent a different inferential scale.

### 2.6. Fragment-level tsRNA profiling using tDrmapper

**Figure 3.**
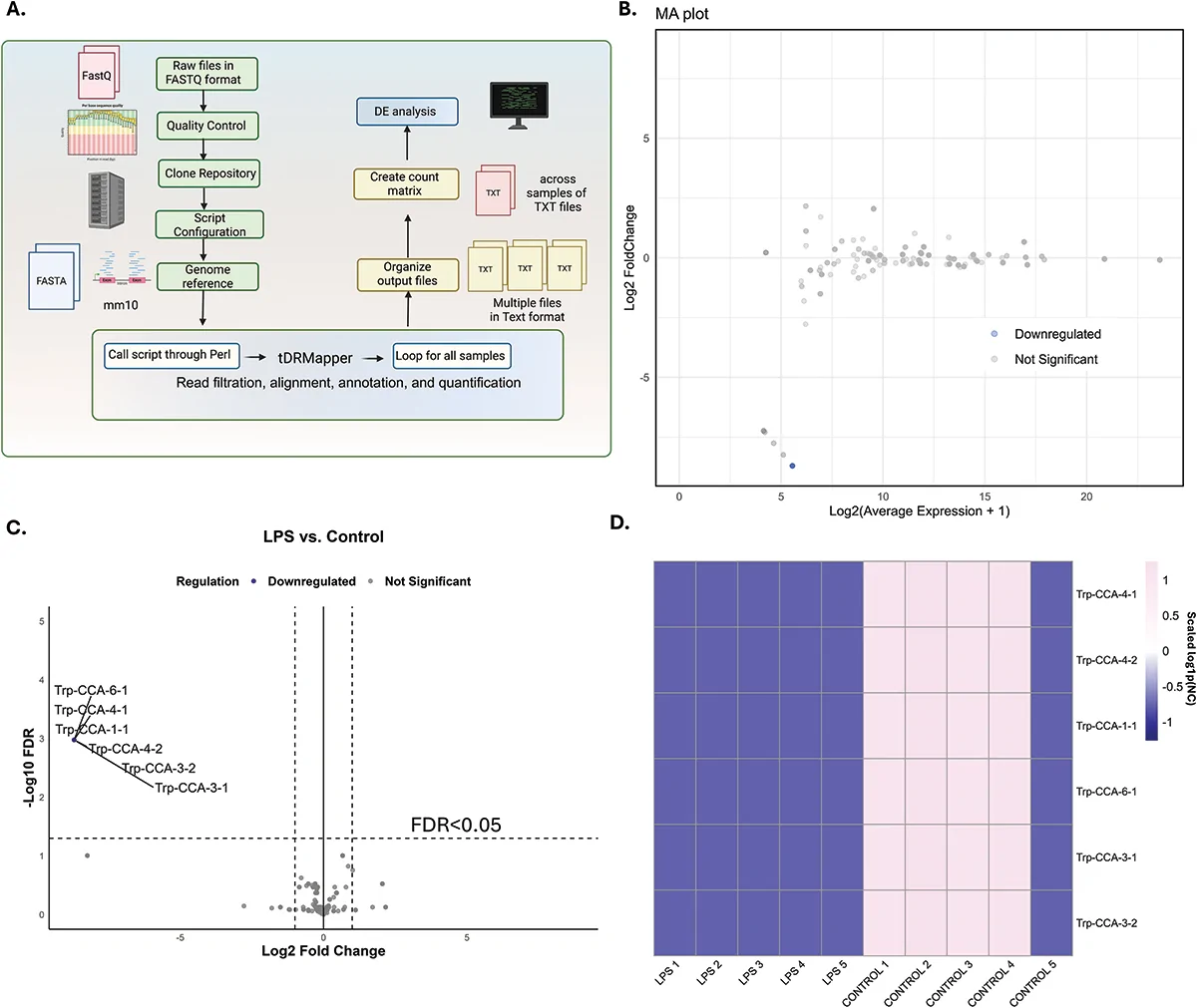
tsRNA analysis using tDRmapper. (A) Schematic workflow illustrating the tDRmapper-based analysis pipeline for tsRNA profiling. Raw sequencing reads in FASTQ format undergo quality control and preprocessing, followed by script configuration and alignment to the reference genome. tDRmapper is executed across all samples to perform read filtration, alignment, annotation, and quantification. Output files generated in text format are organized across samples and compiled into a count matrix, which is subsequently used for downstream differential expression analysis. (B) MA plot showing differential expression of tsRNAs in sperm from adult male offspring of LPS-treated fathers versus saline controls. Each point represents an individual small RNA, plotted as log_2_ fold change versus mean normalized expression. Differentially expressed small RNAs are highlighted. (C) Volcano plot depicting all detected tsRNAs in sperm from adult male offspring of LPS-treated fathers versus saline controls. Log_2_ fold change is shown on the x-axis and −log_10_ false discovery rate (FDR) on the y-axis. The horizontal dashed line denotes an FDR threshold of 0.05. Small RNAs exceeding the significance threshold (FDR < 0.05) are highlighted in dark blue (downregulated) or light pink (upregulated). (D) Heatmap showing expression levels of differentially expressed tsRNAs in sperm from adult male offspring of LPS-treated fathers versus saline controls. Values represent scaled log1p-transformed normalized counts across five biological replicates per group. The color scale indicates relative expression levels. Panel A was created in BioRender. Lu, D. (2026) https://BioRender.com/st5dmyi.

To improve tsRNA resolution, the workflow incorporates tDRmapper, a dedicated tool for mapping and classifying tRNA-derived fragments [36]. tDRmapper addresses tsRNA-specific analytical challenges, including the high sequence redundancy among tRNA gene copies and the structural and sequence similarity that complicate conventional alignment approaches [36]. Adapter-trimmed reads were aligned to curated mature and precursor tRNA reference sequences derived from GtRNAdb (mm10 annotation) using the tDRmapper sequential mapping framework, in which reads are first matched exactly and subsequently evaluated under progressively relaxed mismatch and deletion criteria [36]. Mapped reads are then classified according to their positional origin within the canonical tRNA secondary structure, enabling assignment to biologically interpretable categories such as 5′-derived fragments, 3′-derived fragments, D-loop fragments, anticodon loop fragments, T-loop fragments, and tRNA halves spanning multiple structural domains. Precursor-derived fragments are further annotated based on whether they originate from 5′ leader sequences, 3′ trailer sequences, or the tRNA body [36]. Read counts are recorded at the level of individual tRNA identities and fragment classes, producing sample-resolved count matrices that preserve fragment position, length, and parental tRNA assignment (Figure 3(A)). These class-specific feature-selection criteria restrict downstream testing to defined tRNA-derived fragments. Compared with the broader tRNA identity and end-based annotation categories provided by SPORTS, tDRmapper offers finer resolution across mature and precursor tRNA structures, allowing downstream analyses to distinguish specific fragment classes and region-dependent changes.

### 2.7. piRNA cluster identification

**Figure 4.**
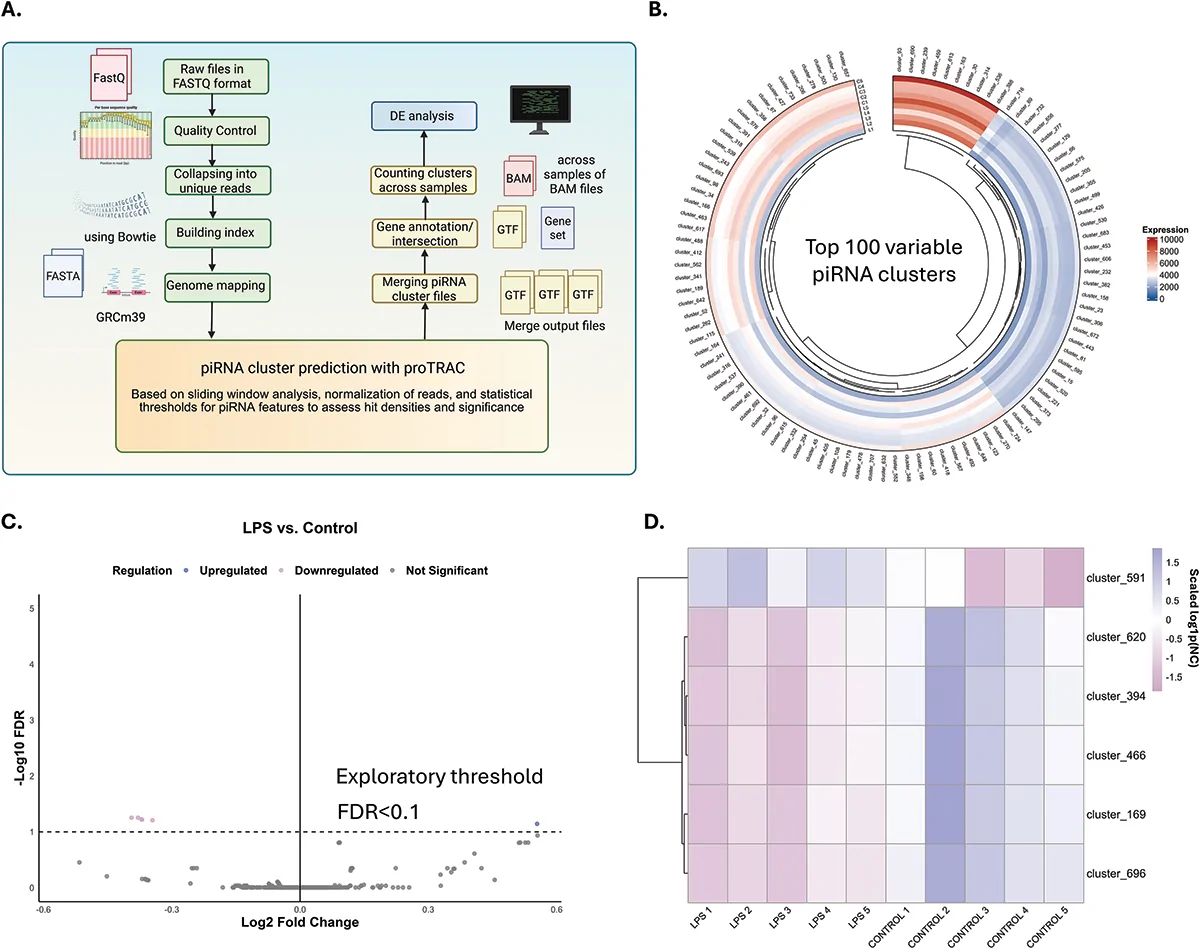
piRNA cluster identification and downstream DE setup. (A) Schematic workflow illustrating the piRNA analysis pipeline based on proTRAC. Raw small RNA sequencing reads in FASTQ format undergo quality control and preprocessing, followed by collapsing into unique reads, index construction, and genome mapping using Bowtie against the reference genome (GRCm39). Mapped reads are used for piRNA cluster prediction with proTRAC, which applies sliding window–based density estimation and statistical thresholds to identify piRNA-enriched genomic regions. Predicted cluster files are merged across samples and intersected with gene annotations as required. piRNA cluster-level read counts are then generated across all samples from BAM files and compiled into a count matrix for downstream differential expression analysis. (B) Circular dendrogram showing top 100 variable predicted piRNA cluster counts across samples. Each branch represents one piRNA cluster, colored according to expression in counts (blue to red indicates 0 to 10,000 counts). (C) Volcano plot depicting all detected piRNA clusters in sperm from adult male offspring of LPS-treated fathers versus saline controls. Log_2_ fold change is shown on the x-axis and −log_10_ false discovery rate (FDR) on the y-axis. The horizontal dashed line denotes an FDR threshold of 0.1. Candidate piRNA clusters passing the exploratory threshold (FDR < 0.1) are highlighted in dark pink (downregulated) or purple (upregulated). (D) Heatmap showing expression levels of candidate piRNA clusters passing the exploratory FDR < 0.1 threshold in sperm from adult male offspring of LPS-treated fathers versus saline controls. Values represent scaled log1p-transformed normalized counts across five biological replicates per group. The color scale indicates relative expression levels. Panel A was created in BioRender. Lu, D. (2026) https://BioRender.com/qtz1fcq.

piRNA analysis was performed using a cluster-centric strategy that reflects the genomic organization and biogenesis logic of piRNA production. After adapter trimming and application of the same quality control procedures described above, reads were first collapsed into unique sequences to reduce redundancy and improve computational efficiency while preserving read abundance information. Collapsed reads were then aligned to the reference genome (GRCm39) using Bowtie version 1.3.1 [34]. Genome indexes were generated once and reused across analysis to ensure standardized mapping across samples. Genome-aligned reads were subsequently processed by proTRAC for *de novo* piRNA cluster prediction [37]. proTRAC identifies candidate clusters by scanning the genome for regions enriched in piRNA-sized reads and evaluating defining features including normalized read density, strand bias, enrichment for 1U or 10A sequence signatures, the proportion of reads within the typical piRNA length range, and the normalized-hits/total-hits ratio used to account for mapping multiplicity [37]. Using a sliding window framework and statistical enrichment thresholds, contiguous genomic windows satisfying these criteria were merged into predicted piRNA clusters on a per-sample basis. Cluster predictions, including genomic coordinates, strand information, and supporting piRNA features, were exported as GTF files. These per-sample cluster annotations were subsequently merged across biological replicates to define a unified candidate piRNA cluster space under a common reference build. As illustrated in Figure 4(A), this workflow emphasizes biologically informed, *de novo* identification of piRNA-producing loci while preserving explicit intermediate outputs linking collapsed reads, genome alignments, and cluster predictions, enabling transparent tracking and reproducible downstream analysis. The top 100 most variable clusters were visualized in a circular heatmap using circlize (v0.4.17) [38] (Figure 4(B)).

### 2.8. Locus-aware piRNA cluster harmonization and differential analysis

To enable cross-sample inference of piRNA activity, we implemented a custom downstream framework that harmonizes per-sample piRNA cluster predictions, defines stable locus-level units of quantification, and supports differential analysis under explicit biological constraints. For cross-sample comparison, cluster GTF files generated by proTRAC from individual samples were concatenated into a single merged annotation and converted into a BED-style coordinate reference where each row represents one predicted piRNA cluster interval. Since identical cluster identifiers can recur across samples, clusters were assigned stable IDs based on row order to ensure one-to-one correspondence between cluster IDs and genomic coordinates. Quantification was performed by intersecting each sample’s BAM file with the merged cluster BED and counting overlapping reads per cluster after length filtering to the piRNA window (21–33 nt), thereby minimizing contamination from non-piRNA fragments. Cluster-level counts across all samples were assembled into a count matrix and used as input for differential expression testing with DESeq2. For workflow validation, when no piRNA clusters met the conventional FDR threshold of 0.05, a prespecified exploratory threshold of FDR < 0.1 was additionally examined because variability introduced by per-sample cluster prediction, overlapping genomic intervals, and multi-mapping reads can reduce statistical sensitivity at the cluster level. This moderately relaxed threshold increased sensitivity to reproducible candidate loci while retaining explicit control of the false discovery rate, with resulting clusters interpreted as exploratory rather than definitive findings. Detailed cluster-level features, including genomic coordinates, mean piRNA-hit counts, log2 fold changes, adjusted *p* values, and overlapping or nearby genes, were compiled for candidate clusters. Cluster intervals were intersected with genomic annotations to identify annotated genes or loci located within or proximal to each cluster. piRNA-sized reads were also converted to BED format and intersected with cluster intervals to enumerate the individual sequences contributing to each cluster, enabling sequence-level follow-up analyses where required.

Cluster quantification was based on the number of piRNA-sized reads overlapping each predicted cluster. These counts provide a practical estimate for comparing clusters between samples but should not be interpreted as direct or fully normalized measures of cluster activity. Quantification may be affected by reads that map to multiple genomic locations and by clusters with partially overlapping boundaries. In addition, merging cluster predictions across samples may generate fragmented or redundant intervals, increasing the number of tested features and reducing statistical power. To complement the quantitative analysis, we implemented a condition-specific interval comparison to identify clusters detected exclusively in either group and reveal genomic differences that may not be captured by differential testing of the merged cluster reference.

### 2.9. Condition-specific non-overlapping piRNA cluster analysis

**Figure 5.**
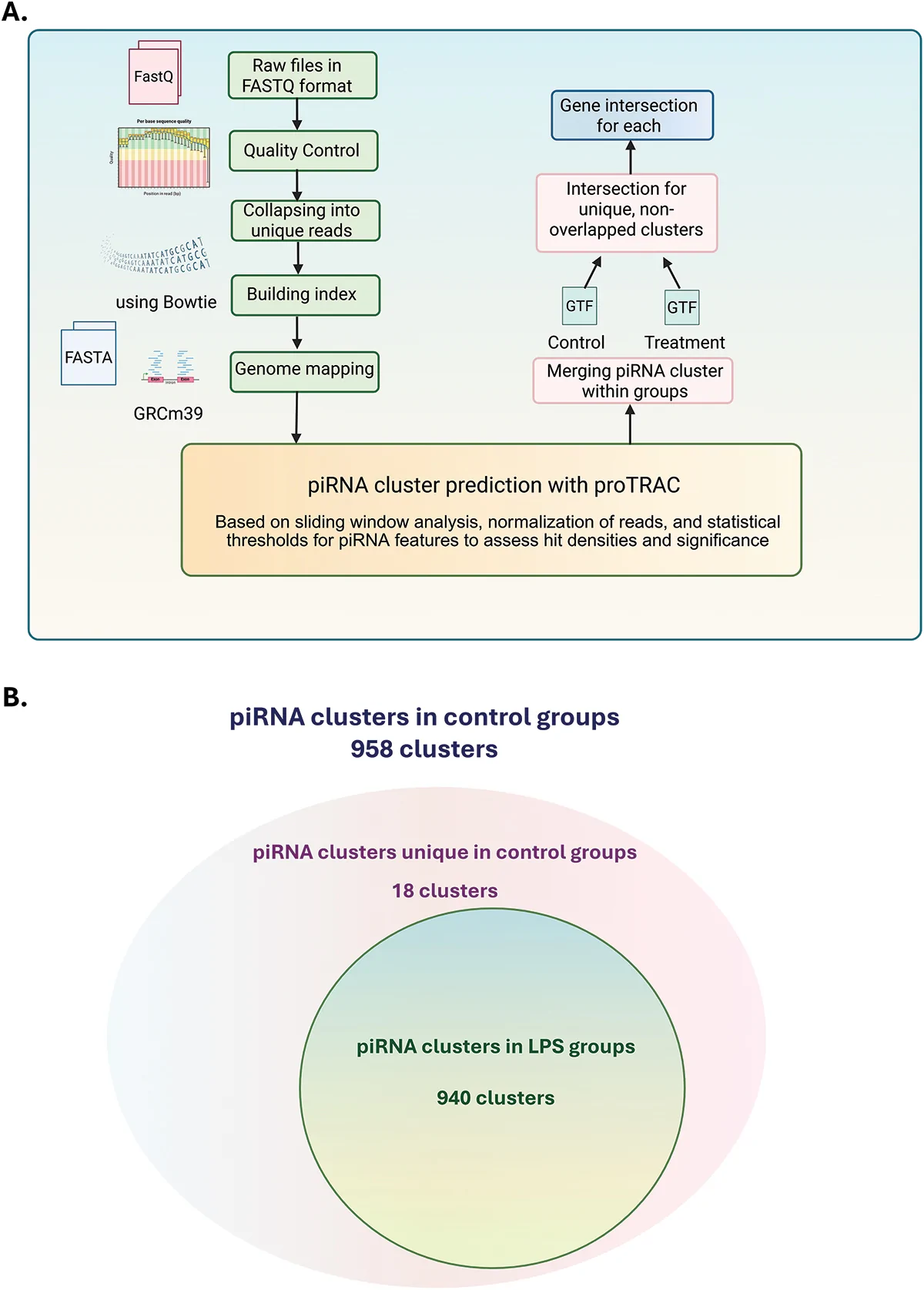
Condition-specific piRNA cluster delineation using non-overlapping genomic intervals. (A) Schematic overview of the piRNA cluster analysis pipeline. Raw small RNA sequencing reads in FASTQ format undergo quality control, collapsing into unique reads, index construction, and genome mapping to the mouse reference genome (GRCm39) using Bowtie. piRNA clusters are predicted independently for each sample using proTRAC. Predicted clusters are then merged separately within control and LPS groups, and group-level cluster sets are intersected to identify unique, non-overlapping piRNA clusters specific to each condition, followed by gene annotation intersection. (B) Venn diagram illustrating the overlap of piRNA clusters between control and LPS groups. A total of 958 piRNA clusters were identified in control samples and 940 in LPS samples, with 18 clusters uniquely present in the control group and absent from the LPS group. Panel A was created in BioRender. Lu, D. (2026) https://BioRender.com/yaejvy6.

To complement cluster-level differential expression analysis and enable biologically constrained interpretation of piRNA activity, we implemented a condition-specific interval comparison to identify piRNA loci detected exclusively in either the LPS or control group and to capture genomic differences that may not be represented by differential testing of the merged cluster reference. Following per-sample piRNA cluster prediction by proTRAC, cluster GTF outputs were merged separately within each condition to generate group level cluster sets for LPS and Control samples. These files were then converted to BED format with strand information retained, coordinate-sorted, and compared using strict interval-overlap criteria to distinguish shared loci from condition-specific loci (Figure 5(A)). For exploratory functional insight, condition-specific cluster intervals were intersected with GRCm39 gene annotations using strand-aware overlap. Gene symbols were then collapsed by cluster to generate locus-level tables reporting chromosome, start and end coordinates, strand, and overlapping annotated genes. These annotations provide preliminary functional context for condition-specific clusters and support prioritization of candidate loci for follow-up analysis.

## 3. Results

### 3.1. Integrated reference-based small RNA profiling

Integrated-reference analysis provided an initial overview of small RNA abundance and differential regulation across all annotated classes. The workflow combined quality-controlled preprocessing, alignment to a composite small RNA reference, feature-level counting, low-count filtering, and DESeq2-based testing (Figure 1(A)). At the feature level, the MA and volcano plots showed that the differential-expression signal was concentrated in miRNAs rather than broadly distributed across the small RNA classes (Figure 1(B,C)). Eleven miRNAs were differentially expressed, with seven upregulated and four downregulated in the LPS group relative to controls, while no other annotated small RNA class met the same significance criteria. The heatmap of these differentially expressed miRNAs showed coherent expression patterns and consistent within-group trends across the five biological replicates per condition (Figure 1(D)). Together, these results demonstrate strong technical performance of the integrated-reference analysis and identify a reproducible, miRNA-dominant expression response, with comparatively limited evidence for differential regulation among the other annotated small RNA classes. Application of the updated integrated-reference pipeline to the Kleeman et al. dataset [26] recovered the previously reported differential upregulation of mmu-miR-3471 and additionally identified mmu-miR-5121 as differentially downregulated in SARS samples. The recovery of the previously reported mmu-miR-3471 change together with the additional detection of mmu-miR-5121 demonstrates how the integrated reference can be efficiently updated as new annotations become available, enabling increasingly comprehensive small RNA profiling over time (**Supplementary Figure S1A–B**).

### 3.2. Complementary class-specific small RNA profiling using SPORTS

To determine whether the limited non-miRNA signal reflected the constraints of the integrated reference, miRNAs, tsRNAs, rsRNAs, and YsRNAs were re-quantified separately using SPORTS for comparison. miRNAs again produced the strongest and most internally consistent differential-expression pattern, with multiple features showing clear effect-size separation in the volcano plot and coherent expression patterns across biological replicates in the heatmap (Figure 2(A,B)). Although the direction and magnitude of regulation varied among individual miRNAs, differential expression was more pronounced in miRNAs than in the other small RNA classes, consistent with the miRNA-dominant pattern identified by the integrated-reference analysis. In contrast, tsRNAs exhibited fewer significantly regulated features, and most detected changes were characterized by more modest fold differences and weaker group-level structure in the corresponding heatmap (Figure 2(C,D)). rsRNAs and YsRNAs showed the most limited differential regulation, with most features remaining close to zero fold change in the volcano plot and few separating from the main distribution, while the heatmap displayed comparatively small and less consistent expression differences between LPS and control samples (Figure 2(E,F)). The class-specific analysis therefore confirmed a graded response in which miRNAs carried the clearest treatment-associated signal, tsRNAs showed a weaker response, and rsRNAs and YsRNAs exhibited minimal differential regulation. When applied to the paternal SARS validation dataset, SPORTS identified six differentially expressed miRNAs, whereas no tsRNA or rsRNA features met the significance threshold (**Supplementary Figure S1C–D**).

### 3.3. Fragment-level tsRNA profiling using tDrmapper

To investigate the limited tsRNA signal detected by the integrated-reference analysis beyond the broader tRNA identity and end-based annotations provided by SPORTS, tDRmapper was used to test whether greater positional resolution revealed a clearer tsRNA response. The analysis retained the structural origin of each read by mapping sequences to mature and precursor tRNAs, classifying fragments according to their position within the parental tRNA, and constructing a fragment-resolved count matrix for differential testing (Figure 3(A)). At this finer resolution, the MA and volcano plots identified a discrete set of regulated tsRNA fragments arising from specific parental tRNAs, whereas most detected fragments remained close to the background distribution and showed little separation between groups (Figure 3(B,C)). Importantly, fragments derived from the same tRNA were not treated as interchangeable counts; their 5′, 3′, internal, or precursor-associated positions were retained so that region-specific changes could be evaluated independently. The heatmap showed reproducible fragment-specific expression patterns across the five LPS and five control replicates, with the regulated fragments displaying more coherent group-associated differences than were apparent in the broader SPORTS tsRNA summaries (Figure 3(D)). These findings show that the apparent strength of the tsRNA response depended on analytical resolution: gene-level aggregation captured relatively modest changes, whereas fragment-aware analysis resolved a smaller but more structured set of regulated tsRNA features. In the validation dataset, tDRmapper reproduced Pro-TGG-3-1 as the same differentially expressed tRF reported in the original analysis, with the feature consistently highlighted by the volcano plot, sample heatmap, and MA plot (**Supplementary Figure S2A–C**).

### 3.4. Locus-aware piRNA cluster harmonization and differential analysis

To move beyond individual annotated piRNA sequences, piRNA analysis was performed at the level of genome-defined candidate clusters. The cluster-centric workflow combined read collapsing, alignment to GRCm39, proTRAC-based cluster prediction, cross-sample harmonization, cluster-level read counting, and differential testing, with the 100 most variable harmonized clusters visualized to summarize sample-level variation across the unified cluster space (Figure 4(A,B)). No cluster reached statistical significance at the conventional FDR threshold of 0.05, suggesting that cluster-level differential testing had limited sensitivity for detecting the modest expression differences present in this dataset. To obtain exploratory insight into these potential differences, the prespecified FDR threshold of 0.1 was additionally examined for further investigation. At this threshold, six candidate clusters showed quantitative differences between LPS and control samples, as reflected by their effect sizes and statistical significance in the volcano plot and their coordinated expression patterns in the heatmap (Figure 4(C,D)). Five candidates were located on chromosome 2, overlapped Gm14144, and showed negative log2 fold changes of approximately −0.34 to −0.39. The remaining candidate was located on chromosome 11, was associated with Zfp652, and showed a positive log2 fold change of approximately 0.55. Mean piRNA-hit counts ranged from 57.8 to 159.1 across these candidates, with adjusted *p* values of approximately 0.056–0.072 (Table 1). The cluster-level analysis therefore defined a small set of exploratory loci with consistent directional changes, while the absence of significant clusters at FDR < 0.05 indicates that these loci should be reported as candidates rather than definitive differential-expression findings. In the paternal SARS validation dataset, the differential piRNA-cluster result was unchanged from the published analysis, indicating that the updated workflow preserved the original cluster-level inference.

**Table 1.** Genomic coordinates and gene/locus annotations of candidate piRNA clusters passing the exploratory FDR < 0.1 threshold.

Clusters	Chromosome	Start	End	FDR	Average number of piRNA hits	log_2_ fold change	Annotated genes/loci
cluster_394	chr2	151090512	151104286	0.055829967	150.5	−0.379153988	Gm14144
cluster_696	chr2	151090512	151102794	0.055829967	143.2	−0.39439832	Gm14144
cluster_466	chr2	151090852	151105873	0.059610019	150.5	−0.371477387	Gm14144
cluster_169	chr2	151091238	151103084	0.060405895	142.5	−0.369445835	Gm14144
cluster_620	chr2	151090671	151106878	0.062072223	159.1	−0.345260666	Gm14144
cluster_591	chr11	95655322	95662485	0.071998686	57.8	0.554273632	Zfp652

Table listing the genomic coordinates, FDR values, average number of piRNA hits, log_2_ fold-change, and associated genes for each cluster. *N* = 5 samples for LPS group and *n* = 5 samples for control group.

### 3.5. Condition-specific non-overlapping piRNA cluster analysis

To complement quantitative testing within the harmonized cluster reference, piRNA clusters were also compared as condition-specific genomic intervals. Per-sample proTRAC predictions were merged within the control and LPS groups, followed by strict interval-overlap analysis to distinguish shared loci from clusters detected exclusively in either condition (Figure 5(A)). This analysis identified 958 control clusters and 940 LPS clusters. Under the strict non-overlap definition, eighteen clusters were detected exclusively in the control group, whereas none were detected exclusively in the LPS group. Most LPS clusters were instead contained within, or overlapped, control-cluster intervals rather than forming separate genomic loci (Figure 5(B)). The eighteen control-specific clusters were distributed across multiple chromosomes and included loci with protein-coding, predicted-gene, and miRNA-associated annotations. Their genomic coordinates and overlapping or nearest annotated genes were compiled at cluster level, providing a locus-resolved summary of the control-specific set rather than reducing the comparison to a single cluster count (Table 2). Taken together, the interval and annotation results show that the dominant between-group difference was not the widespread appearance of new LPS-specific piRNA-producing regions, but a small set of control-specific loci within an otherwise largely shared cluster landscape. Applying the added group-overlap analysis to the validation dataset detected 19 clusters in each condition, comprising one control-specific and two SARS-specific loci. The remaining clusters overlapped or partially overlapped between groups (**Supplementary Figure S2D**). The genomic coordinates and associated annotations of the three condition-specific loci are summarized in Supplementary Tables S1 and S2.

**Table 2.** Genomic coordinates and gene/locus annotations associated with the 18 piRNA clusters uniquely identified in control samples.

Cluster_ID	Chromosome	Start	End	Annotated Genes/Loci
cluster_17	chr10	61264602	61269555	Eif4ebp2
cluster_18	chr10	61264755	61268927	Eif4ebp2
cluster_19	chr10	61264814	61270849	Eif4ebp2
cluster_20	chr10	86423028	86429377	NA
cluster_21	chr11	100349541	100353930	Klhl11
cluster_22	chr11	100350235	100354893	Klhl11
cluster_23	chr15	88862090	88866717	Gm8702, Mlc1
cluster_24	chr15	88862584	88866710	Gm8702, Mlc1
cluster_25	chr15	88863090	88866711	Gm8702, Mlc1
cluster_26	chr4	42584684	42590285	Gm52989, ENSMUSG00000121464, Gm2163
cluster_27	chr5	114953841	114959111	ENSMUSG00000133116, Gm57857
cluster_28	chr5	114972326	114992460	Gm13821, ENSMUSG00000131648
cluster_29	chr5	114972715	114992455	Gm13821, ENSMUSG00000131648
cluster_30	chr8	37558263	37573690	NA
cluster_31	chr9	3182026	3188284	4930433N12Rik
cluster_32	chr9	88500848	88505221	ENSMUSG00000131140
cluster_33	chrX	65836202	65857625	Mir471, Mir741, Mir463, Mir880, Mir878, Mir881, Mir871, Mir470, ENSMUSG00000133389
cluster_34	chrX	65869567	65882727	Mir465c-1, Mir465b-1, Mir465c-2, Mir465b-2, Mir465

Listed features include cluster ID, chromosomal location, genomic start and end positions, and overlapping or nearest annotated genes.

## 4. Discussion

This study shows that reproducible sperm small RNA-seq analysis requires class-aware interpretation and cannot rely on a single annotation strategy. Using mouse sperm as a case study, we demonstrate that analytical resolution strongly influences biological interpretation across small RNA classes with distinct biological and analytical properties. miRNA-associated signals were relatively robust under reference-based approaches, whereas tsRNA and piRNA-like signals required more cautious, class-specific treatment because of fragment heterogeneity, sequence redundancy, and genomic cluster organization.

Validation of the updated workflow using the published paternal SARS-CoV-2 sperm small RNA dataset [26] provided an external methodological benchmark. Recovery of the previously reported miRNA, tRF, and piRNA-cluster findings supports analytical continuity with the original implementation, while the additional miRNA and condition-specific piRNA loci identified here indicate broader detection enabled by expanded annotations and newly incorporated modules. The complementary outputs of SPORTS and tDRmapper further underscore the value of class-specific tools operating at different analytical resolutions. These findings suggest that incorporating updated references and class-specific modules can extend analytical coverage while preserving the interpretability of previously established small RNA signals.

A central principle emerging from this workflow is that no single annotation strategy is equally appropriate for all small RNA classes. Integrated reference-based alignment provides a useful overview of annotated small RNA abundance and can identify robust miRNA-associated signals. However, such approaches are less suitable for small RNA classes with high sequence redundancy, fragment heterogeneity, or complex genomic organization. This is particularly relevant for tsRNAs and piRNA-like signals. For these classes, apparent abundance differences may reflect reference structure, read assignment ambiguity, fragment resolution, or genomic multi-mapping instead of straightforward changes in RNA expression. Therefore, class-aware analysis is essential not only for improving detection but also for avoiding overinterpretation.

Another important interpretive consideration is that small RNA-seq read counts represent relative sampling within each sequencing library and do not directly measure absolute RNA abundance. Although our DESeq2 analyses used raw integer counts with size-factor normalization rather than RPM-normalized values, the data remain compositional. Therefore, apparent changes in one sncRNA class may partly reflect changes in the relative contribution of other classes rather than absolute depletion or enrichment of that class [39]. Consistent with this concern, we added a SPORTS-based class-composition analysis to summarize the relative contribution of major sncRNA classes across samples (**Figure S3**). These results should therefore be interpreted as relative within-library changes, particularly in the absence of spike-ins, northern blot anchoring, or other independent approaches for absolute quantification [39].

The comparison between SPORTS and tDRmapper illustrates why tRNA-derived small RNA analysis benefits from fragment-level resolution. SPORTS provides a unified framework for annotating small RNA reads across multiple reference databases, including miRNAs, rsRNAs, tsRNAs, piRNAs, and additional non-coding RNA families from Ensembl and Rfam [32,33]. This integrated approach enables comprehensive compositional profiling while minimizing double-counting of reads across functionally distinct RNA biotypes, making it well-suited for exploratory analysis and cross-class comparisons. Although SPORTS provides robust gene- and anticodon-level summaries of tRNA-derived reads, it does not resolve fragment-specific distinctions arising from the same parental tRNA. Consistent with its cross-class design philosophy, SPORTS emphasizes compositional integration rather than structural resolution. In contrast, tDRmapper captures fragment-level positional information within mature and precursor tRNAs, enabling discrimination between 5′ fragments, 3′ fragments, loop-derived fragments, and tRNA halves [36].

These methodological differences affected the resulting biological interpretation. SPORTS detected modest condition-associated tsRNA differences in the paternal LPS model, but these signals showed limited structural organization. In contrast, tDRmapper revealed fragment-specific regulatory patterns with greater biological specificity, consistent with evidence that tRFs arise through cleavage at distinct positions and can exhibit fragment-specific biological properties [6–8]. Moreover, tRF cleavage positions and relative abundance vary substantially across biological contexts, supporting the need for analysis beyond parental tRNA identity alone [9]. Together, these observations suggest that tsRNA biology operates at a resolution finer than the parental tRNA identity captured by multi-class frameworks. Accordingly, tsRNA interpretation benefits from analytical tools capable of resolving fragment-level distinctions. Importantly, this does not diminish the value of SPORTS for integrated small RNA profiling; rather, it highlights the complementary roles of multi-class compositional frameworks and class-specific, high-resolution analytical tools.

The piRNA cluster analysis requires particularly cautious interpretation. piRNAs are defined by their association with PIWI proteins, and total small RNA-seq data cannot definitively establish PIWI binding. Therefore, the genome-based analysis presented here should be interpreted as candidate piRNA-enriched signals rather than validated PIWI-bound piRNA expression. Within this limitation, the workflow provides two conceptually distinct downstream strategies that address complementary biological questions. The first approach, locus-aware cluster harmonization followed by differential expression testing, treats piRNA clusters as stable genomic features with variable activity levels, analogous to gene-level differential expression. By merging per-sample cluster predictions into a unified reference annotation and quantifying read density across samples, this framework enables statistical inference on cluster activity changes in response to experimental treatments. This approach successfully identified candidate piRNA loci with consistent quantitative shifts between LPS and control groups, providing interpretable outputs compatible with established genomic workflows. However, the differential expression paradigm carries implicit assumptions that may not fully align with piRNA biology. Merged cluster annotations can introduce fragmented or redundant intervals when cluster boundaries differ slightly across samples, inflating the feature space and reducing statistical power. More fundamentally, this approach presumes that all detected clusters exist in both conditions and that regulation operates primarily through quantitative modulation of preexisting loci, potentially obscuring condition-dependent initiation or silencing of piRNA production from specific genomic regions. The second approach, condition-specific non-overlapping cluster identification, relaxes these assumptions by treating cluster presence itself as a biologically informative variable. Instead of merging clusters across all samples, this strategy consolidates predictions within each experimental group and applies strict interval overlap logic to classify clusters as shared, control-specific, or treatment-specific. This design directly addresses the biological question of whether environmental or physiological perturbations can trigger *de novo* piRNA biogenesis from previously silent loci or suppress production from constitutively active regions.

The preference for condition-specific discovery over differential expression analysis in piRNA analysis is grounded in both biological and statistical considerations. Biologically, piRNAs are generated from discrete genomic clusters whose transcriptional activation is developmentally regulated and controlled by epigenetic mechanisms [40], making binary presence-absence a meaningful biological state distinct from graded expression differences. Statistically, the condition-specific framework avoids multiple testing penalties associated with fragmented merged annotations and provides interpretable outputs without requiring complex normalization across genomic intervals of variable length and composition. Furthermore, clusters identified as condition-specific under non-overlap criteria represent high-confidence candidates for functional validation, as their detection is contingent on reproducible signal across biological replicates within one group and complete absence in the other, filtering out stochastic or low-abundance noise. In contrast, differential expression analysis operates on individual piRNA hits within each cluster, not aggregate cluster-level expression. Condition-specific discovery instead evaluates cluster activity as a biological entity, making it the recommended approach for identifying categorical changes in cluster expression states. Thus, the two approaches address distinct but complementary dimensions of piRNA regulation rather than serving as interchangeable alternatives, with condition-specific discovery providing a more direct and statistically robust framework for identifying candidate cluster-level activation or suppression states associated with biological perturbation.

Several limitations should be considered when interpreting this workflow and case study. The workflow integrates and standardizes established tools and does not introduce a new primary small RNA discovery algorithm. Its contribution is therefore practical and interpretive, providing a reproducible structure for class-aware analysis, documenting key analytical decision points, and adding custom downstream modules for candidate cluster harmonization and overlap-based interpretation. The case study also used pooled sperm samples, which supports group-level molecular profiling but prevents direct individual-level association between sperm small RNA variation and offspring phenotypes. This limitation is particularly relevant for downstream molecular–phenotypic modeling, which requires matched molecular and behavioral data from the same father–offspring units. In addition, candidate piRNA-enriched cluster analysis from total small RNA-seq cannot substitute for PIWI-immunoprecipitated small RNA sequencing or other approaches that directly validate PIWI association. Annotation-dependent analysis also remains sensitive to reference choice, database completeness, and mapping parameters, especially for miRNA database selection, tsRNA fragment classification, and piRNA-like signal interpretation.

Although rsRNAs were included in the integrated-reference and SPORTS analyses, the observed differential signal was limited and the current annotations primarily represented parental rRNA or reference-level features rather than position-resolved fragments. In addition, validated target-prediction frameworks for rRNA-derived fragments remain less established than those for miRNAs. Future extensions could incorporate species-compatible fragment-level resources, such as MINRbase, to support more detailed functional investigation of nuclear and mitochondrial rRNA-derived fragments [41]. Additional non-coding RNA classes, including long non-coding RNAs (lncRNAs), could be incorporated in future extensions as suitable annotations and class-specific resources become available, given their reported roles in gene regulation [42]. These considerations highlight the importance of treating the workflow as a reproducible framework for transparent exploratory analysis and candidate prioritization.

Library-preparation chemistry is another important consideration when interpreting the present dataset. The sperm small RNA-seq data analyzed here were generated using a conventional small RNA library-preparation approach and therefore represent the fraction of sperm sncRNAs captured by this method, rather than the broader modified sncRNA landscape. This is particularly relevant for tsRNAs and rsRNAs, which may carry terminal or internal RNA modifications that interfere with adapter ligation or reverse transcription. Emerging modification-aware approaches, such as PANDORA-seq and CPA-seq, would be valuable in future studies to improve the detection of modified sncRNA populations and complement conventionally prepared small RNA-seq datasets [43,44]. A recent sperm-aging study using PANDORA-seq further supports this point by identifying age-associated tsRNA and rsRNA changes that were not readily detected by conventional small RNA-seq [45].

Another further extension of this workflow is the integration of small RNA profiles with matched phenotypic datasets. As outlined conceptually in **Figure S4**, candidate small RNA features could be paired with behavioral, metabolic, or physiological readouts using sparse feature-selection approaches followed by nonlinear modeling to prioritize molecular features associated with complex offspring phenotypes. Behavioral readouts from multiple assays could first be grouped by domain, such as anxiety-like, cognitive, depressive-like, and social phenotypes, and standardized using Z-score normalization, following established cross-assay integration approaches [26]. Variance-stabilized small RNA count matrices could then serve as high-dimensional molecular predictors. For each behavioral domain, LASSO regression could be applied independently to identify sparse domain-associated candidate features while reducing multicollinearity. The resulting domain-associated feature sets could then be incorporated into Random Forest or related nonlinear models, together with genomic annotation features where appropriate, to explore feature importance and potential interactions across behavioral domains. However, this type of analysis requires individual-level matching between molecular and phenotypic data. In the present study, sperm small RNA libraries were generated from pooled samples from three males per biological replicate, whereas behavioral phenotypes were not directly matched to the same individual males contributing to each molecular replicate. Therefore, formal molecular–phenotypic prediction was not performed here. Future studies using matched father–offspring designs could apply this framework to evaluate associations between sperm small RNA variation and offspring outcomes.

Overall, this study highlights the importance of analytical caution and class-aware interpretation in small RNA-seq analysis. Although mouse sperm from a paternal immune activation model was used as the case study, the analytical principles apply more broadly to small RNA profiles from other tissues and biological contexts. Different small RNA classes vary substantially in their annotation reliability, biological interpretation, and appropriate analytical resolution. miRNA-associated signals can be captured using reference-based approaches, tsRNA interpretation benefits from fragment-level mapping, and piRNA-like signals from total small RNA-seq require cautious treatment as candidate piRNA-enriched cluster-associated signals rather than definitive PIWI-bound piRNA measurements. By making these analytical assumptions explicit, the workflow helps prioritize candidate small RNA features and genomic regions for future functional validation in studies of paternal environmental exposures and intergenerational epigenetic mechanisms.

## 5. Conclusion

In conclusion, this study presents a modular and containerized class-aware workflow for reproducible small RNA-seq analysis across multiple RNA classes. By combining standardized preprocessing, complementary class-specific modules, and explicit reporting of analytical limitations, the workflow provides a practical resource for small RNA analysis across diverse tissues and biological contexts. Mouse sperm was used as a biologically relevant case study, while the modular structure supports future extension to improved reference resources, PIWI-bound datasets, additional small RNA species, and matched molecular–phenotypic study designs.

## Supplementary Material

Figure S2.png

Figure S1.png

Figure S3.png

Figure S4.png

Supplemental Figure 1 260731.docx

## Data Availability

The small RNA sequencing datasets used in this study are deposited in the NCBI Sequence Read Archive (SRA) under BioProject accession PRJNA1502842. The supplementary file containing the FUSION family-level results for the paternal LPS and SARS-CoV-2 sperm small-RNA datasets is deposited to https://doi.org/10.6084/m9.figshare.33102323.

## Article highlights

- Using mouse sperm as a case study, a modular, containerized workflow enables reproducible, class-aware analysis of small RNA-seq data.
- Integrated-reference analysis supports broad miRNA and rsRNA profiling, while dedicated modules preserve tsRNA fragment and piRNA-cluster resolution.
- Fragment-level analysis identifies structured tsRNA changes that may be obscured by parental tRNA-level aggregation.
- Locus-aware and condition-specific analyses prioritize candidate piRNA-enriched regions while supporting cautious interpretation.
- Complementary modules support feature-, fragment-, and cluster-level small RNA analysis.

## Code availability

GitHub: https://github.com/dlld975/Small-non-coding-RNA-analysis-pipeline.

Operating system: Linux (tested under Ubuntu 20.04 and later; compatible with WSL2 environments).
