# Supplementary material for: A modular class-aware workflow for small RNA sequencing analysis using mouse sperm as a case study
Source: Epigenomics. 2026 Sep 6;18(9):1071–86. doi: 10.1080/17501911.2026.2725425 (PMC13613897; doi:10.1080/17501911.2026.2725425)
Supplement: Supplementary file 1 [file IEPI_A_2725425_SM2986.docx]

**Figure S1.** **External validation of miRNA differential-expression results using the published SARS-CoV-2 sperm small RNA dataset.** (A) Volcano plot from the updated integrated reference-based analysis comparing SARS and control samples. Each point represents an annotated miRNA; dashed vertical lines indicate the fold-change cutoff and the dashed horizontal line indicates FDR < 0.05. The analysis recovered mmu-miR-3471 as significantly upregulated and identified mmu-miR-5121 as significantly downregulated, showing clear separation from the bulk of non-significant miRNAs. (B) Heatmap of scaled log1p-normalized counts for the significant miRNAs identified by the integrated reference workflow, showing opposite condition-associated expression patterns for mmu-miR-5121 and mmu-miR-3471 across SARS and control samples. (C) Volcano plot from SPORTS-based miRNA profiling of the same validation dataset. SPORTS identified six differentially expressed miRNAs, including downregulated mmu-miR-5121 and mmu-miR-345 and upregulated mmu-miR-7013. (D) Heatmap of scaled log1p-normalized counts for SPORTS-detected miRNAs, showing coordinated expression differences between SARS and control samples. Together, these analyses support reproducible miRNA-level signal detection across independent annotation strategies, whereas no tsRNA or rsRNA features met the significance threshold in the SPORTS validation analysis.

**Figure S2. External validation of tsRNA and piRNA-cluster analyses using the published SARS-CoV-2 sperm small RNA dataset.** (A) Volcano plot from tDRmapper-based tRF analysis comparing SARS and control samples. Pro-TGG-3-1 was the only differentially expressed tRF and showed a large positive fold change in SARS samples. (B) Heatmap of scaled log1p-normalized counts for Pro-TGG-3-1 across individual SARS and control samples, illustrating the sample-level expression pattern underlying the differential signal. (C) MA plot for the tDRmapper analysis, highlighting Pro-TGG-3-1 as an outlying upregulated tRF relative to the overall distribution of detected tRF features. (D) Venn diagram summarizing group-level piRNA-cluster overlap after applying the condition-specific cluster analysis. Nineteen piRNA clusters were detected in each condition, with one control-specific cluster, two SARS-specific clusters, and the remaining clusters overlapping or partially overlapping between groups.

| **Cluster_ID** | **Chromosome** | **Start** | **End** | **Annotated Genes/Loci** |
| --- | --- | --- | --- | --- |
| **cluster_1** | **chr17** | **23764637** | **23769479** | **ENSMUSG00000140489, ENSMUSG00000140395,**  **6330415G19Rik** |

**Table S1.** **Control-specific candidate piRNA-enriched cluster in the paternal SARS-CoV-2 validation dataset.** Genomic coordinates and nearby gene/locus annotations for the candidate cluster on chromosome 17 identified exclusively in the mock-control group by strict interval-overlap analysis.

| **Cluster_ID** | **Chromosome** | **Start** | **End** | **Annotated Genes/Loci** |
| --- | --- | --- | --- | --- |
| **cluster_2** | **chr10** | **76215821** | **76218041** | **Pcnt** |
| **cluster_3** | **chr13** | **21342582** | **21354456** | **NA** |

**Table S2.** **SARS-specific candidate piRNA-enriched clusters in the paternal SARS-CoV-2 validation dataset.** Genomic coordinates and nearby gene/locus annotations for the two candidate clusters on chromosomes 10 and 13 identified exclusively in the SARS-CoV-2 group by strict interval-overlap analysis. NA indicates that no nearby annotated gene or locus was identified.

**Figure S3.** **Relative contribution of major sncRNA classes across sperm small RNA-seq libraries.** Stacked bar plot showing the percentage contribution of miRNAs, tRNA-derived small RNAs (tsRNAs), rRNA-derived small RNAs (rsRNAs), YRNA-derived small RNAs (YsRNAs), piRNA-like reads, and other/ambiguous annotations in sperm samples from adult male offspring of LPS-treated fathers (LPS 1–LPS 5) and saline controls (C1–C5). Class proportions were calculated from the SPORTS per-sequence annotation output by assigning each collapsed sequence to one non-overlapping class and summing reads per class. Values are expressed as percentages of total clean SPORTS output reads per sample. The dashed line separates LPS and control samples. These values represent relative within-library composition and should not be interpreted as absolute RNA abundance. An exact permutation test of centered log-ratio-transformed class proportions yielded a pseudo-F statistic of 6.2494 and P = 0.0317 based on 252 possible group-label assignments.

**Figure S4.** **Machine learning–based feature selection and modeling.** Behavioral phenotypes were grouped by domain and standardized to z scores. For each behavioral domain, piRNA cluster–level counts were used as predictors and behavioral z scores as responses in LASSO regression to perform domain-specific feature selection, yielding separate sets of piRNA clusters associated with anxiety-like, cognitive, and depressive-like behaviors. Selected clusters from all domains were then combined and incorporated into random forest models together with genomic annotation features to enable nonlinear classification and estimation of cluster importance. Final piRNA clusters were prioritized based on feature importance scores exceeding a predefined threshold across behavioral domains.
